# Triticum vulgare extract exerts an anti-inflammatory action in two *in vitro* models of inflammation in microglial cells

**DOI:** 10.1371/journal.pone.0197493

**Published:** 2018-06-14

**Authors:** Luca Sanguigno, Antonella Casamassa, Niccola Funel, Massimiliano Minale, Rodolfo Riccio, Salvatore Riccio, Francesca Boscia, Paola Brancaccio, Luca Emanuele Pollina, Serenella Anzilotti, Gianfranco Di Renzo, Ornella Cuomo

**Affiliations:** 1 Department of Molecular Medicine and Medical Biotechnology, School of Medicine, Federico II University of Naples, Naples, Italy; 2 Division of Pharmacology, Department of Neuroscience, Reproductive and Dentistry Sciences, School of Medicine, Federico II University of Naples, Naples, Italy; 3 Department of Translational Research and The New Technologies in Medicine and Surgery, University of Pisa, Italy; 4 Farmaceutici Damor S.p.A, Naples, Italy; 5 Second Division of Surgical Pathology, Hospital of Pisa, Italy; 6 IRCSS SDN, Naples, Italy; University of Cologne, GERMANY

## Abstract

Triticum vulgare has been extensively used in traditional medicine thanks to its properties of accelerating tissue repair. The specific extract of Triticum vulgare manufactured by Farmaceutici Damor (TVE-DAMOR) is already present in some pharmaceutical formulations used in the treatment of decubitus ulcers, skin lesions and burns. It has been recently suggested that this Triticum vulgare extract may possess potential anti-inflammatory properties. In the light of these premises the aim of the present paper was to verify the anti-inflammatory role of TVE, using the LPS-stimulated microglia model of inflammation. In particular the effect of different concentrations of TVE on the release of several mediators of inflammation such as nitric oxide, IL-6, PGE2 and TNF alpha was evaluated. More important, the anti-inflammatory effect of TVE was confirmed also in primary rat microglia cultures. The results of the present study show that TVE exerts anti-inflammatory properties since it reduces the release of all the evaluated markers of inflammation, such as NO, IL6, TNF alpha and PGE2 in LPS-activated BV2 microglial cells. Intriguingly, TVE reduced microglia activation and NO release also in primary microglia. Indeed, to verify the pathway of modulation of the inflammatory markers reported above, we found that TVE restores the cytoplasmic expression of p65 protein, kwown as specific marker associated with activation of inflammatory response. The evidence for an inhibitory activity on inflammation of this specific extract of Triticum vulgare may open the way to the possibility of a therapeutical use of the Triticum vulgare extract as an anti-inflammatory compound in certain pathological states such as burns, decubitus ulcers, folliculitis and inflammation of peripheral nerve.

## Introduction

Triticum vulgare has been extensively used in traditional medicine thanks to its properties of accelerating tissue repair. A specific aqueous extract of Triticum vulgare (TVE-DAMOR) manufactured by Farmaceutici DAMOR (Naples, Italy) is currently an active component in the manufacture of certain pharmaceutical products already marketed in Italy and abroad under the brand name Fitostimoline^®^, in the formulation of cream and medicated gauze and is commonly used for the treatment of decubitus ulcers, burns, scarring delays, dystrophic diseases, and in conditions in which it is necessary to stimulate re-epithelialization or tissue regeneration processes. In fact, it has been reported that the active component of Fitostimoline^®^ products (TVE) determines a marked acceleration of tissue repairing processes, stimulates chemotaxis and the fibroblastic maturation, and significantly increases the fibroblastic index, which are crucial points in the repairing processes [1–4].

It has been suggested that Triticum vulgare extract may possess potential anti-inflammatory properties. Thus, it has demonstrated that TVE reduces the carrageenin-induced hind-paw edema of rats in a dose-dependent manner and that this effect is not related to the secretion of adrenal steroids, as this activity was also present in adrenalectomized rats. Furthermore, in a more recent double-blind randomized controlled trial, it has been shown that Fitostimoline^®^ may be useful in the treatment of vaginal inflammation and vulvar dystrophy [5].

In the light of these premises, the aim of the present study was to verify the possible anti-inflammatory role of TVE, using the Lipopolysaccharide (LPS)-stimulated microglia model of inflammation [6–7]. To better clarify the effects of TVE we used two different cell culture models exposed to LPS: the murine BV2 microglial cells and the primary rat microglia cultures. In particular, the release of several mediators of inflammation such as nitric oxide, IL-6, PGE2, TNFalpha and NF-kB p65 subunit as well as the activation of microglia by Iba1 expression levels were evaluated in the LPS-stimulated microglia cultures pre-incubated with different concentration of TVE.

## Materials and methods

### Plant description

*Triticum vulgare*, the binomial scientific name of a plant of Graminaceae family, is the commonly known wheat plant. It is grown under controlled conditions in the laboratory of Farmaceutici DAMOR, Naples, Italy. The voucher specimen is DF/237/2014 and it is deposited in the herbarium of the Medical Botany Chain of University of Salerno, Italy. The commercially available seeds are purchased from Consorzio Agrario Lombardo Veneto from Northern Italy. The batch number for the seeds used for the present paper was 12/001-B10148/201/04.

#### Triticum vulgare extract

TVE-DAMOR is a specific aqueous extract of Triticum vulgare, obtained by a complex and specific process as already described [8]. It was a gift of Farmaceutici Damor (Naples, Italy).

#### Cell cultures

The immortalized murine BV2 cell line (ICLC ATL03001 Interlab Cell Line Collection, Banca Biologica e Cell Factory, Italy) was cultured in Dulbecco’s Modified Eagle’s Medium (DMEM, Invitrogen) supplemented with 10% fetal bovine serum (FBS), 1% penicillin-streptomycin (Invitrogen), and 1% glutamine (Invitrogen). Cultures were grown at 37 °C in 5% CO2 until 80% confluence. In order to perform the treatments and analyses, cells were split when they reached confluence using trypsin/EDTA solution in PBS. We used two different modality of seeding according the different molecular determination investigated below: *1) For NO and Pro-Inflammatory determinations*. BV2 mouse microglial cells were seeded in 12-well plates, in order to obtain three different experiments for each concentration of TVE. The mediums were harvested for analyses described as follows. *2) To analyze NF-kB p65 subunit*. BV2 mouse microglial cells were seeded in 8-well Chamber Slides (CS) (Lab-Tek^®^ Chamber Slide^™^ system, Nalge Nunc International, Naperville, IL, US), putting in 5000 cells/well in a 650 μL final volume. CS were prepared in order to obtain three different experiment in triplicate. After treatments, cells were fixed directly on the slides by Carnoy’s solution for 10 min and the chamber slide wells were removed by mechanical support following manufacturer’s instructions. The immunofluorescence (IF) for NF-kB p65 subunit detection was performed as described in immunofluorescence section.

#### Primary rat microglia cultures

Cultures of primary rat microglia were prepared from primary rat mixed glial cell cultures, as previously described [9,10]. In brief, cerebral cortices isolated from 1- to 2-days-old-rats were first dissociated enzymatically in a solution containing 0.125% trypsin and 1.5 mg/mL DNase (Sigma Aldrich, St. Louis, MO, USA) and then mechanically in Dulbecco’s modified Eagle’s medium supplemented with 10% fetal bovine serum, 100 U/mL penicillin, 100 μg/mL streptomycin, and 2 mM L-glutamine (Gibco, Milan, Italy). The pellets were re-suspended and plated in tissue culture flasks in normal medium at 37°C in a humidified, 5% CO_2_ incubator. After 7–9 days of culture, once confluent, the primary microglia were separated by mechanical shaking of flasks on a rotary shaker for 60 min at 200 r.p.m. and plated onto 12-wells plate poly-d-lysine (Sigma-Aldrich, Milan, Italy)-coated coverslips. This procedure yields 98% Iba1-positive cells.

### *In vitro* inflammatory stimuli and TVE treatments

BV2 cells and rat primary microglial cells were seeded in 12-wells plate. Cells were pre-treated with TVE. The BV2 cell line were exposed to the following concentration: 1–2.5–5 and 10% of TVE while, the primary microglia cultures were pre-incubated only with 10% TVE. Twelve hours after TVE pre-stimulation, immortalized and primary microglia cells were incubated with the Lipopolisaccharide (LPS, Sigma) inflammatory stimulus (100 ng/mL) for 24 hours.

#### Confocal immunofluorescence studies

Primary microglia cell cultures were fixed in 4% paraformaldehyde for 30 minutes. After blocking, cells were incubated with the primary polyclonal antibody (rabbit anti-Iba1 1:500, Wako) for 24h. Subsequently, microglial cells were incubated with the fluorescent-labeled secondary antibody Alexa 488-conjugated anti-rabbit. Cell nuclei were counterstained with the fluorescent DNA-binding dye Hoechst-33258. Images were observed with a Zeiss LSM 700, laser scanning confocal microscope. Single images were taken with an optical thickness of 0.7 μm and a resolution of 1024×1024.

Iba1 fluorescence intensity in primary microglia cell cultures were quantified in pixel intensity by the NIH image software [11]. All images were obtained with an ×40 objective. Experiments were performed at least three times and at least 6 images from each group were analysed. Data were expressed as a percentage of control group.

#### Definition of microglia activation

Primary microglial cells were defined as activated based on the following criteria: a) there was a significant increase in expression of Iba1 microglial marker; b) there was a significant increase in NO production.

#### Determination of NO production

To assess NO production, the accumulation of nitrite was measured in the culture medium of BV2 mouse and primary microglia cell cultures by using GRIESS assay [12]. The culture medium was mixed with a solution of α-naphthyl-ethilen-diamine (0.1% solution w/v in H_2_O) and sulphanilamide (1% solution w/v, in 5% H_3_PO_4_) in the ratio 1:1 for 10 min and kept in dark conditions. The production of nitrite was recorded spectrophotometrically by monitoring the absorbance at 540nm. A standard curve of NaNO_2_ was used to quantify the production of nitrites obtained during the experiments. NO production was expressed as percentage of control group. Experiments were performed at least three times and in triplicate.

#### Determination of PGE2 and pro-inflammatory cytokines IL-6 and TNF alpha

PGE2, IL-6 and TNF alpha concentration were assessed in cell supernatants with commercially available kits. Cellular mediums were centrifuged at 4000 rpm for 5 min. Levels of PGE2 in the media were measured by the enzyme immunoassay (EIA) (Assay Designs Inc., Ann Arbor, MI, USA) according to the manufacturer’s instructions. Pro-inflammatory cytokines (TNFalfa and IL-6) were determined using ELISA kits (Mouse TNF alpha Elisa kit, Thermo Scientific Pierce Protein Research Products and Mouse IL6 Elisa kit, Thermo Scientific Pierce Protein Research Products) according to the manufacturer’s instructions. Experiments were performed at least three times and in triplicate.

#### Determination of nuclear concentration of NF-kB p65 subunit by Immunofluorence

The quantification of NF-κB p65 subunit in BV2 cells was performed by immunofluorescence (IF) assay. After fixation slides were rinsed in PBS1X for 10 min. In order to detect p65 subunit we used a polyclonal antibody, (1:100; 1h at RT; NF-κB p65 (D14E12) XP^®^ Rabbit mAb #8242, Cell Signaling Techonologies, Leiden, The Netherlands). After washing, the fluorescent secondary antibody was applied (1:50; 30 min at RT in darkeness; Anti-rabbit IgG Fab2 AlexaFlour 488, #4412S, Molecular Probes, Cell Signaling Techonologies, Leiden, The Netherlands). The nuclei counterstaining was performed using a special fluorescence antifade containing DAPI (ProLong^®^ Gold Antifade Reagent with DAPI #8961, Molecular Probes Cell Signaling Techonologies, Leiden, The Netherlands).

Samples were stored at 4°C until observation. The visualization, nuclear migration and quantification of NF-κB p65 subunit was performed using a confocal microscope (AXIO vert 200, Zeiss, Weztlar, Germany) and its dedicated software for imaging acquisition and digital imaging process (AXIOvision version 4.2.3.1, Zeiss, Weztlar, Germany). The images were acquired at 40X magnification. Five different images (DAPI, Green, Merge (M), Bright field (BF) and BF+M), were acquired for each field as well as we reported in the corresponding figure. To quantify the signal of each color channel (Blue and Green), we draw a vector up to merge images in order to obtain a graph reporting the intensity of fluorescence (IF) looking at IF signals in both cytoplasms and Nnuclei. The vector analyzed 40 different points/cell, across nucleus and cytoplasm. The length of vector was equal to 6 μm. The intensity of aqua spectrum was calculated by the ratio (IF R) between IF vales obtained by blue channel out of those obtained by green channel. Nuclear protein expression of p65 was associated in presence of aqua color. The range of aqua spectrum ranged as follow: 1.00<IF R<1.40. Indeed, we overlap the BF images and M images, to verify the location of aqua color inside the nuclei.

### Statistical analysis

Experiments were performed at least three times and the data are expressed as the mean ± SEM of the values obtained in three separate experiments. Statistical comparisons between controls and treated groups were performed by one-way analysis of variance followed by Newman-Keuls’s test. p < 0.05 was considered significant.

## Results

### TVE reduces NO production in BV2 microglia cells exposed to LPS stimulation

TVE significantly reduced the release of nitrite at concentrations of 2.5, 5 and 10%, when preincubated for 12 hours before LPS treatment compared to cells exposed to LPS alone.

In particular, while LPS induced a significant increase in NO release compared to control conditions, NO production by LPS-stimulated BV2 cells pretreated with TVE at concentrations from 2.5 to 10% were similar to those of control cells not exposed to LPS. These findings indicates that these TVE concentrations were able to abolish the pro-inflammatory, LPS-mediated, release of NO.

**Fig 1:**
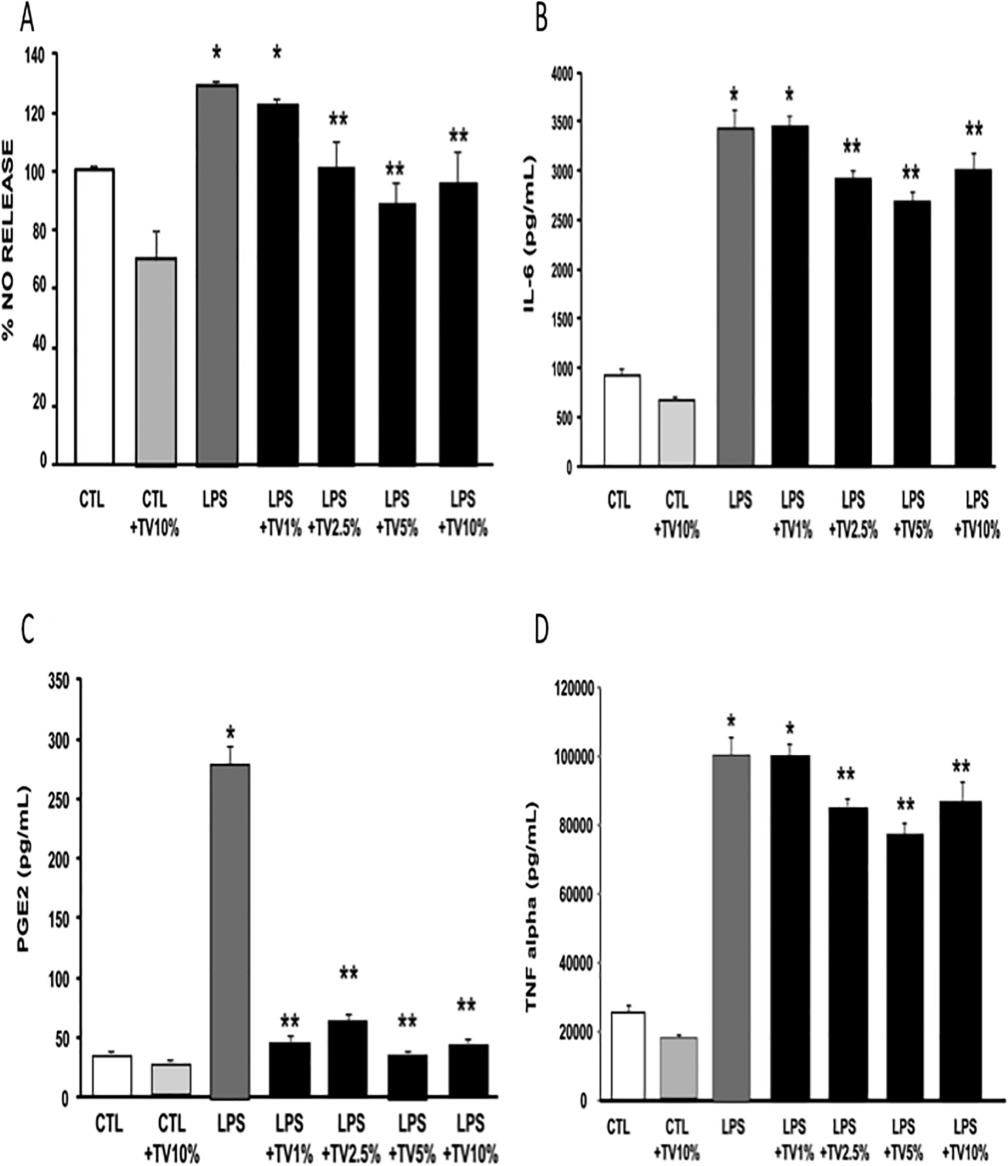
Effect of TVE on NO, IL-6, PGE2 and TNFα release in BV2 cells. BV2 cells were exposed to TVE for 12 hours at different concentrations (1-2.5-5-10%). LPS was added 12 hours after TVE pre-incubations. The release was evaluated 24 hours after LPS exposure. Data were expressed as percentage of target release with respect to untreated cells (100%). N = 6 for each experimental group. * p<0.05 *vs* control non-treated cells; ** p<0.05 *vs* LPS treated cells. Graphs represented the results for the following markers: **A)** NO; **B)** IL-6; **C)** PGE2 and **D)** TNFα. All concentrations were expressed as (pg/mL).

By contrast, 1% TVE did not determine any significant reduction of nitrite release compared to cells exposed to LPS alone. Moreover, 10% TVE was able to reduce also the basal release of nitrites in BV2 cells not exposed to LPS (Fig 1A). In fact, under these experimental conditions NO release was significantly lower than that observed in control group.

### TVE reduces PGE2 production in BV2 microglia cells exposed to LPS stimulation

TVE reduced the production of PGE2 from LPS-activated BV2 microglia cells. In particular, LPS induced a significant increase in PGE2 release compared to control cells. Interestingly, cells preincubated twelve hours with TVE at all the tested concentrations and then exposed to LPS showed a significant reduction in the release of PGE2 compared to LPS treated group (Fig 1B).

### TVE reduced IL-6 production in BV2 microglia cells exposed to LPS stimulation

TVE preincubation significantly reduced the production of IL-6 in LPS-activated BV2 microglia cells. In particular, while LPS induced a strong increase in IL-6 release, BV2 cells preincubated with TVE at concentrations from 2.5 to 10% and then exposed to LPS showed a reduction in the release of IL-6 measured 24 hours after LPS stimulation compared to LPS-treated cells. Moreover, 10% TVE slightly reduce also the basal release of IL-6 in BV2 cells not exposed to LPS (Fig 1C).

### TVE reduces TNF alpha production in BV2 microglia cells exposed to LPS stimulation

TVE reduced the production of TNF alpha from LPS-activated BV2 microglia cells. In particular, LPS induced a significant increase in TNF alpha release compared to control cells. Interestingly, cells preincubated overnight with TVE and then exposed to LPS showed a significant reduction in the release of TNF alpha measured 24 hours after LPS stimulus compared to LPS treated group (Fig 1D).

### TVE restores cytoplasmic expression of NF-kB p65 protein and reduces its nuclear expression in BV2 cells after LPS exposure

**Fig 2:**
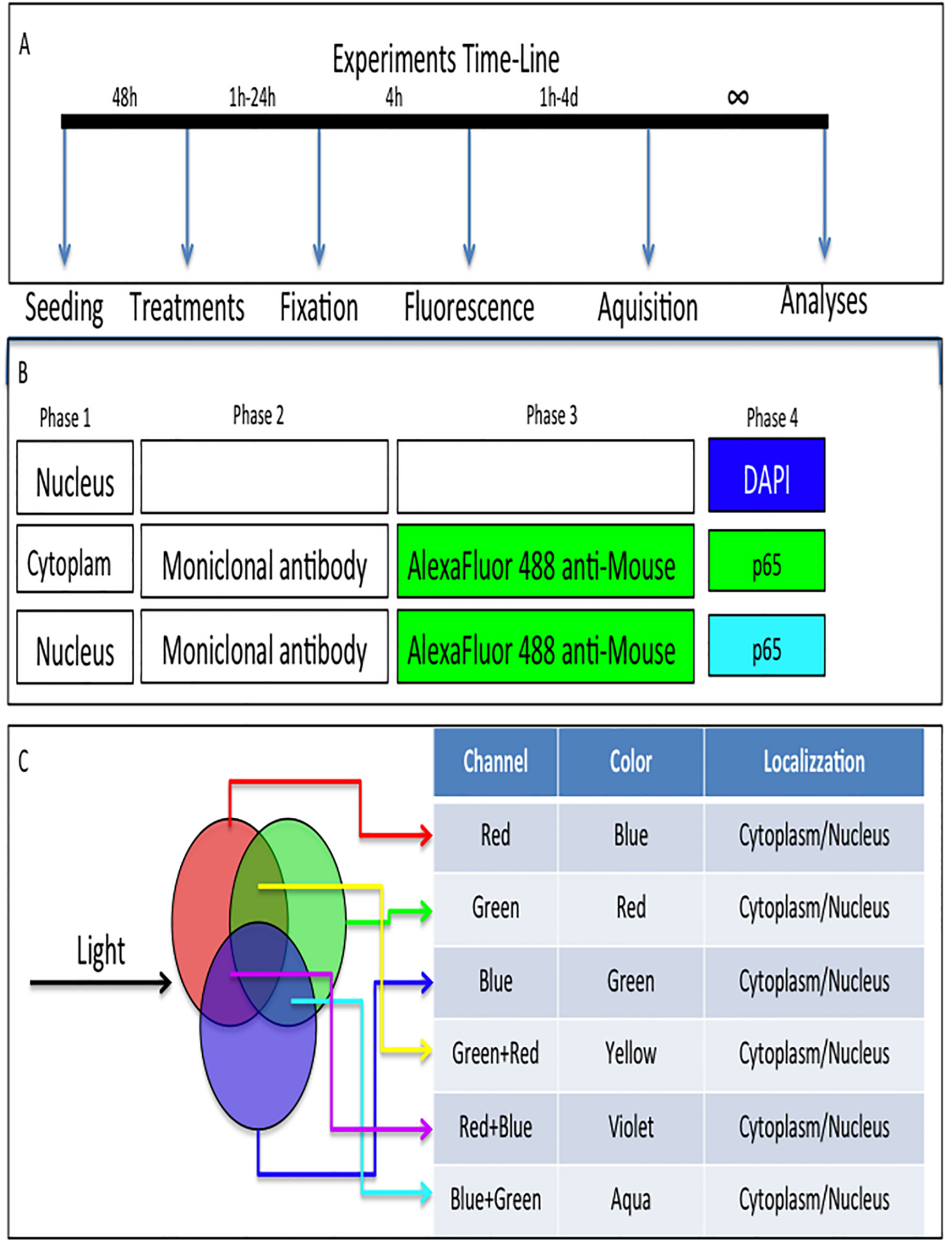
Flow chart of Immunoflurescence (IF). **A)** Time line of experiments showing the different actions, starting from the seeding of the cells to analyses of fluorescence.**B)** Immunoflurescence procedure. We used an Indirect IF using primary and secondary antibodies. Nuclei were counterstained by DAPI. **C)** Spectra of color. Three primary color (Red, Blue and Green) and three additional merged colors (Violet, Yellow and Aqua) were detectable by confocal system. Colors and their cellular localization are report in the table.

**Fig 3:**
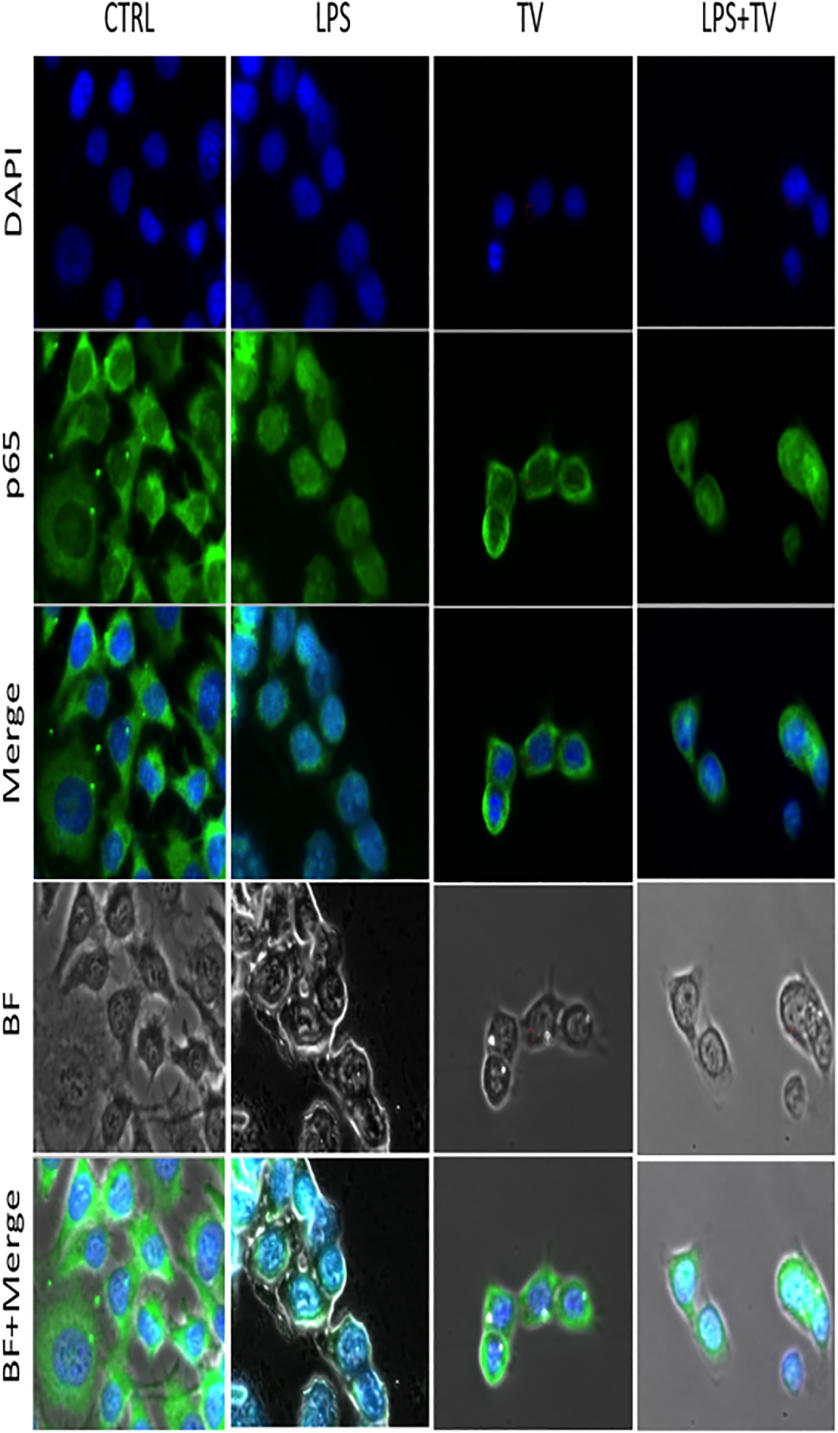
Down-regulation of nuclear expression of NF-kB p65 subunit LPS-stimulated BV2 microglial cells. BV2 cells were stimulated with 100 ng/ml LPS as described before. Both nuclear and cytoplasmic localizations of p65 ware evaluated using anti-p65 polyclonal antibody and a FITC-labelled anti-rabbit IgG antibody (Green color, lines 2). DAPI was used in order to identify nuclei (Blue color, line 1). Images of cells were obtained by bright field light (BF, line 4) and UV light excitation lines 1,2 and 3). Nuclear translocation of p65 subunit is visible by detection of Aqua color (Merge, line 3). Cytoplasmic p65 expression was associated with different intensity of green color inside the cytoplasm. To visualize better the Aqua color co-localized into the nuclei, we overlapped the BF images upon merge of fluorescence (line 5). The bars reported different combinations of treatments in BV2 cells as follows: CTRL (LPS-/TVE10%-), LPS (LPS+/TVE10%), TV (LPS-/TV10%+) and LPS+TV (LPS+/TV10%+). The clear Aqua color was present only in LPS treated cells. No Aqua color significant differences were observed comparing CTRL *vs* TV and LPS+TV colums.

**Fig 4:**
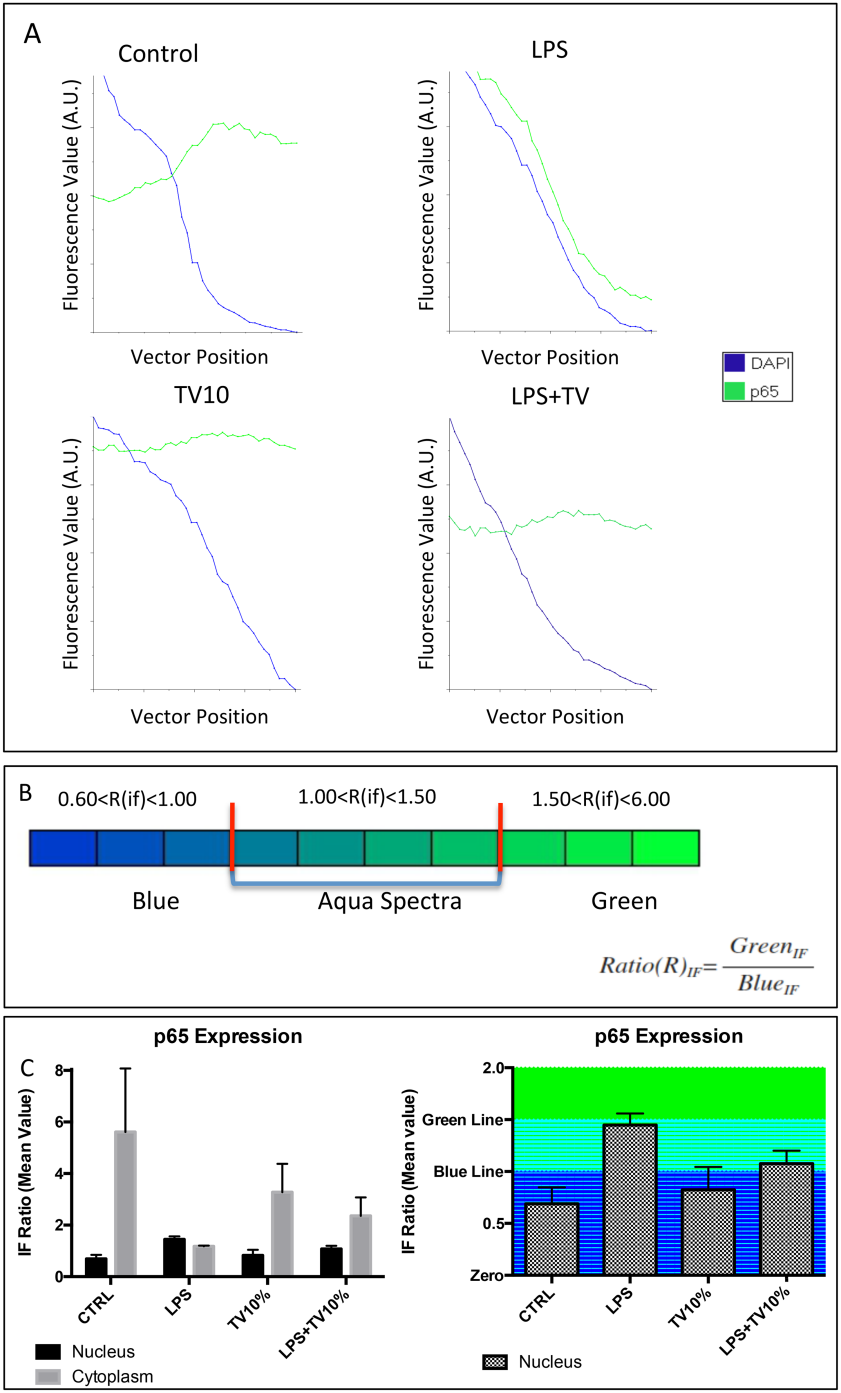
Nuclear and cytoplasmic of p65. **A)** IF levels obtained by analyzing merge images reported in Fig 3 line 3. Graphs reported the intensity of IF from both green and blue colors. The cross-match between the blue line (DAPI) and green line (p65) highlight the nucleus/cytoplasm borderline. Notably, the IF lines cross-match is remarkable in all treatment except in which we used only LPS. Indeed, in LPS experiments, the IF green intensity was often higher with respect to the blue one, revealing an higher IF ratio. **B)** Colorimetric representation of aqua spectrum (left) and the formula used in order to obtain the mathematic value of fluorescence ratio (right). **C)** Quantification of both cytoplasmic and nuclear expression of p65 protein. IF ratio associated with nuclear and cytoplasmic expression of p65 were obtained graphs interpretation reported in fig 4A. The values showed significant statistical differences (p<0.001). In particular, we would like catch the attention of the reader, looking at our result in in LPS experiments. The ratio between nucleus and cytoplasm is very close to 1.0 value. It means that p65 is very similar in both cellular compartment. **D)** Nuclear quantification of p65 associated with aqua spectrum value. Higher details were observed looking at p65 nuclear expression only. Combining IF value analyses and the range of aqua spectrum calculation, we obtained directly the differences in therms of p65 nuclear expression in all treatment. LPS exerted a significant difference in comparison with the other treatments (p<0.001). Looking at LPS+TV10% treatment an IF levels associated with aqua spectrum were observed, but no significant differences were found comparing this treatment *vs* CTRL.

TVE modulated nuclear migration of NF-kB p65 subunit. Indeed, the p65 subunit nuclear translocation were analyzed by fluorescence microscopy (Fig 2). Images reporting Blu color (DAPI), Green color (p65), aqua color (Merge) were assessed after LPS+/TVE10%-, LPS-/TVE10%+ and LPS+/TVE10%+ treatments. Untreated BV-2 BV2 cells (LPS-/TVE10%-) were used as control. The fluorescence intensity (IF) of acqua color (merge between Blue and Green) revealed nuclear translocation of p65. LPS stimulation caused the movement of NF-KB p65 subunit from the cytoplasm into the nucleus (Fig 3) whereas the presence of TVE10%, with or without LPS restored its cytoplasmic expression (Fig 4C). To further verify p65 nuclear expression, IF value data (Fig 3) were analyzed by the software. Our results showed that TVE downregulated p65 nuclear localization induced by LPS (Fig 4C).

### Triticum vulgare extract alleviated LPS-induced activation in rat primary microglia cultures

**Fig 5:**
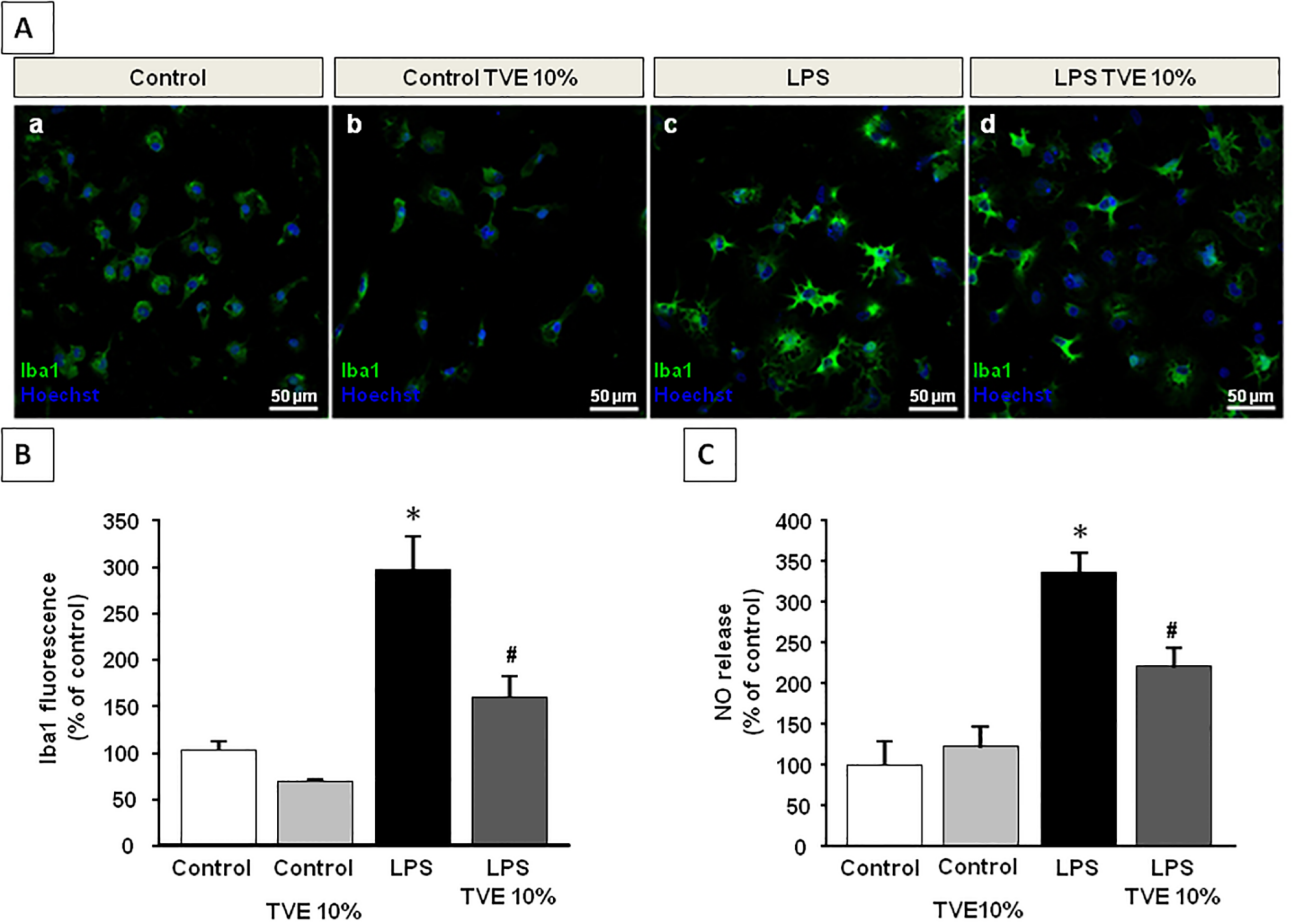
Effects of TVE on LPS-stimulated primary microglia. **A**. Immunofluorescence images displaying Iba1 (green) immunoreactivity of primary microglia under control condition (a), incubated with 10% of TVE (b), LPS-stimulated (c) and LPS-stimulated microglial cells pre-incubated with 10% of TVE (d). Cell nuclei were counterstained with the fluorescent DNA-binding dye Hoechst (blu) Scale bars: 50 μm. **B**. Quantification of Iba1 fluorescence intensities in primary microglia. The values represent the mean ± SEM (n = 6–8). *p < 0.05 *versus* controls, ^#^p< 0.05 *versus* LPS. **C**. NO production in primary microglia. Abbreviations: LPS Lipopolysaccharide, TVE Triticum Vulgare Extract, SEM Standard Error of the Mean.

Confocal and quantitative studies of Iba1 expression levels revealed TVE ability to alleviate LPS-induced activation in primary rat microglia. Single immunolabeled experiments performed with anti-Iba1 antibody in primary microglia showed that 10% TVE pre-incubation for 12 hours before LPS treatment, was able to induce a significantly reduction of Iba1 expression compared to LPS exposure alone (Fig 5A).

Furthermore, quantitative fluorescence experiments revealed that TVE incubation in primary microglial cells did not determine any significant modulation in Iba1 fluorescence intensity if compared with control condition. Interestingly, TVE pre-incubation in LPS-exposed microglia was able to prevent the strongly increase of Iba1 expression levels observed in LPS-stimulated primary microglia (Fig 5B).

To further confirm the ability of TVE to reduce LPS-induced activation we also carried out NO production assay by Griess reaction. This assay revealed that 10% TVE was able to abolish the pro-inflammatory, LPS-mediated, release of NO performed in primary microglia cultures. In this condition, NO values were similar to those observed in control cells not exposed to LPS. As expected, LPS induced a significant increase in NO release compared to control cells. Accordingly with the results obtained in BV2 cell line, a significantly reduction in NO release was observed in LPS-microglia cells pre-treated with 10% TVE if compared with LPS-exposed alone (Fig 5C).

## Discussion

The results of the present study show that the extract of Triticum vulgare (TVE) manufactured by Damor Farmaceutici exerts anti-inflammatory properties since it reduces the release of specific markers of inflammation, such as NO, IL6, TNF alpha and PGE2 in LPS-activated BV2 microglial cells, that represent a widely accepted model of neuroinflammation, as previously described [13–14]. Additionally, TVE restored the cytoplasmic levels of p65, reducing its nuclear expression. Indeed p65 cytoplasmic localization is commonly associated with regulation of inflammatory pathways [6].

Microglia, when activated, can be potent immune effector cells in CNS. In this regard, microglial cells are now considered to be the most important component of the brain immune system [14–15].

During neuroinflammation, microglia are activated, change their morphology, and release various cytotoxic mediators, such as nitric oxide (NO), tumour necrosis factor-alpha (TNFα), interleukin-1Beta (IL-1β) and prostaglandin E2 (PGE2). This effect is due to the activation of NF-kB triggered by phosphorylation, subsequent degradation of inhibitor of kB (IkB) and nuclear migration of p65 subunit, also in BV2 cells model [7]. This process subsequently leads to translocation of free NF-kappaB protein (p65) to the nucleus where it promotes the expression of pro-inflammatory genes such as the pro-inflammatory cytokines (TNFα, IL-6 and IL-1β), cyclooxygenase-2 (COX-2), and inducible nitric oxide synthase (iNOS) [6,16]. Our results, showing that the increase in nuclear expression of p65 after LPS treatment was reverted in presence of 10% TVE strongly support the anti-inflammatory action of TVE. It has to be underlined that we used a quantitative methodology in order to demonstrate the impact of p65 in our model [7]. More interestingly, our results in primary rat microglia cultures strongly support the anti-inflammatory role of TVE. Indeed, since microglial activation is associated with the increase of Iba1 microglial marker expression [17, 18] we performed immunofluorescence experiments for Iba1 marker in order to evaluate TVE effects on microglia activation. Our results showed that the strong activation of Iba1 induced by LPS was reverted by TVE treatment. Accordingly, the increase in NO release after LPS was prevented by TVE incubation, thus confirming the anti-inflammatory effect of TVE also in a primary microglia model.

As regard as the possible mechanism of this effect, it should be mentioned that this specific extract of Triticum vulgare is able to promote tissue regeneration by promoting the proliferation of fibroblasts and the remodeling phase [19–20] and that the two processes, inflammation and tissue regeneration, are strongly connected [21]. In fact, during the processes of tissue regeneration healing of wounds and skin lesions, remodeling processes involving the activation of fibroblasts and other connective tissue cells such as macrophages and dendritic cells are activated [22–29]. Furthermore, the regeneration process is finely regulated by a balance of cellular mediators such as cytokines, chemokines etc. which regulate the state of activation and stimulation of the different cellular components. The same mediators and cellular components are involved also in the regulation of inflammatory processes [30]. Indeed, when a tissue is damaged, inflammatory processes are activated and followed by the process of healing after the removal of the source of damage and inflammation. Dendritic cells, macrophages and fibroblasts, together with mediators such as cytokines and chemokines [31–32] are involved in the transition between the state of the phase of inflammation and tissue regeneration. Mediators such as IL-1, TNFα, IL-6, NO, and PGE2, that are activators of inflammation, usually decrease in order to facilitate the process of tissue repair and remodeling as a result of the damage suffered [33–34].

It is well known that the specific extract of Triticum vulgare produced by Damor Farmaceutici acts on fibroblast activating and promoting the process of tissue repair and wound healing. In the present paper we showed that TVE is able to reduce the expression levels of the well known agents triggering the inflammatory process, such as IL-6, TNFα, prostaglandin E2 and nitric oxide in LPS-stimulated BV2 microglial cells and in primary rat microglia, thus exerting an anti-inflammatory activity and favoring the transition from the inflammatory to regenerative process.

## Conclusions

Overall, the results of the present study support the evidence for an inhibitory activity on inflammation of this specific extract of Triticum vulgare, and may open the way to the possibility of a therapeutical use of the TVE as an anti-inflammatory compound in certain pathological states such as burns, decubitus ulcers, folliculitis and inflammation of peripheral nerve.
